# Treatment of Paederus Dermatitis with Sambucus ebulus Lotion

**Published:** 2014

**Authors:** Mohammad Ali Ebrahimzadeh, Mohammad Reza Rafati, Maryam Damchi, Mosoud Golpur, Fatemeh Fathiazad

**Affiliations:** a *Pharmaceutical Sciences Research Center, School of Pharmacy, Mazandaran University of Medical Sciences, Sari, Iran. *; b *Pharmaceutical Sciences Research Center, Department of Clinical Pharmacy, School of Pharmacy, Mazandaran University of Medical Sciences, Sari, Iran. *; c *Department of Dermatology, School of Medicine, Mazandaran University of Medical Sciences, Sari, Iran. *; d *Department of Pharmacognosy, *S*chool of Pharmacy, Tabriz University of Medical Sciences, Tabriz, Iran. *

**Keywords:** Paederus dermatitis, *Sambucus ebulus*, Inflammation, Erythema, Itching

## Abstract

Paederus dermatitis is an irritant contact dermatitis due to accidental contact by a beetle belonging to the genus paederus. In this study, clinical efficacies of *S. ebulus *fruit extract solution in patients affected by paederus dermatitis were evaluated. A randomized double-blind, prospective, placebo-controlled clinical trial was performed in 62 patients with clinical symptoms and sings of dermatitis due to paederus beetles. The patients received either a topical solution of palemolin (a 5% S. ebulus fruit extract in ethanol 70%) or ethanol 70% topical solution thrice a day. Topical hydrocortisone ointment was prescribed for all patients. Palemolin was statistically more effective in controlling of burning, pain, inflammation, drying the wound, infections and acceleration of healing than control group (p ≤ 0.05). Specially in controlling of inflammation, palemolin had more significant efficacy (p < 0.001) than control group. About 63.6% of patients in palemolin group cured during first 24 h (versus 27.4% in control groups). The problems related to lesions in 93.9% of patients were eliminated completely during 48 hours after the beginning of the treatment by palemolin (versus 65.4% in control groups). Topical 5% solution of *S. ebulus *fruit extract is an effective pharmaceutical preparation in treatment of paederus dermatitis.

## Introduction

**Figure 1:**
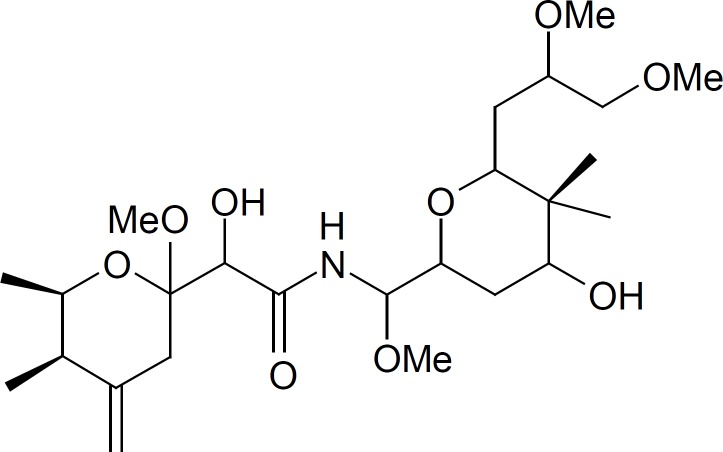
Structure of Paederin

Paederus dermatitis, also known as dermatitis linearis is a peculiar irritant contact dermatitis characterized by erythematous and bullous lesions of sudden onset on exposed areas of the body. The disease is provoked by an insect belonging to the genus *Paederus*. This genus belongs to family *Staphyllinidae*, order *Coleoptae*, class *Insecta *and consists of over 622 species which are distributed worldwide (1). This beetle does not bite or sting, but accidental brushing against or crushing the beetle over the skin provokes the release of a fluid which contains paederin (Figure 1), a potent vesicant (2). Paederin is an amide with two tetrahydropyran rings and makes up approximately 0.025% of an insect᾽s weight. Genes responsible for the biosynthesis of this compound has been reported (3). 

Adults of these beetles are usually 7-10 mm long and 0.5 mm wide. They have a black head, lower abdomen and elytra and a red thorax and upper abdomen (4). Paederin is not produced by the beetles themselves. It is synthesized in only about 90% of the females (5). Larvae and males only store paederin acquired maternally or by ingestion. It is vesicant and blocks mitosis by inhibiting protein and DNA synthesis without affecting RNA synthesis (6). Paederus dermatitis was reported in many countries including Australia (7), Malaysia (8), Nigeria (4), Central Africa, Uganda, Okinawa, Sierra Leone (6), Argentina, Brazil, France, Venezuela, Ecuador and India (9) (10), Iraq (11), and Turkey (12). One hundred and fifty-six cases of paederus dermatitis were also reported among patients attending a dermatology clinic in northern Iran from May to October 2001 (1). 

The dermatitis may affect persons of either sex, all ages, races or social conditions. The exposed areas are affected with a greater frequency. The lesions are erythematous and edematous which may be linear, giving a whiplash appearance. The vesicles generally appear towards the center of the plaque. The vesicles turn into pustules quite frequently. The signs appear after 24 to 48 h of contact and take a week or more to disappear (4, 13). Complications include post inflammatory hyperpigmentation, secondary infections, and extensive exfoliating and ulcerating dermatitis requiring hospitalization (1, 6, 7). Ocular and genital involvement is relatively common; it occurs secondary to transfer of the toxic chemical from elsewhere on the skin by Fingers (1). Recently, it has been demonstrated that the production of paederin relies on the activities of an endosymbiont (*Pseudomonas *species) within Paederus. The manufacture of paederin is largely confined to adult female beetles. Larvae and males only store paederin acquired maternally (*i.e*., through eggs) or by ingestion. It is vesicant and blocks mitosis at levels as low as 1 ng/ mL apparently by inhibiting protein and DNA synthesis without affecting RNA synthesis. Acantholysis is probably caused by the release of epidermal proteases (13). 

Washing the skin immediately with soap and water can prevent linear dermatitis or more severe symptoms (14). The acute vesicular lesions heal completely in 10-12 days, with a transitory post-inflammatory hyperchromic patch. In severe cases, it can cause neuralgia, artheralgia, fever and vomiting. Erythema may persist for months in severe cases it can cause man-hours loss in productivity and school absence (14, 15). Application of cold wet compresses followed by topical steroid and antibiotic, if secondarily infected, is recommended (6). 

*Sambucus ebulus *(Caprifoliaceous), (Common name: Dwarf elder, Danewort, it is locally called: Palem, Peilam) extensively growth in the northern regions of Iran (16, 17). Flavonoids, steroids, tannins, glycosides, cardiac glycosides, caffeic acid derivatives, ebulitins and volatile substances of these species were previously reported (18). Iranian traditional medicine uses the leaves, fruits and rhizomes of *S. ebulus *in treating some inflammatory cases such as, bee and nettle bites, arthritis and sore-throat (16, 19, 20). In addition, it has been reported to be anti-bacterial, antioxidant, convenient in treatment of burns and infectious wounds, edema, eczema, urticaria, inflammation and rheumatism and was examined as *against Jack-bean urease by Berthelot alkaline phenol-hypochlorite method for *Helicobacter pylori infection (21-24). These effects gave us a solid basis to make use of *S. ebulus *in treatment of paederus dermatitis. This study was designed to compare the clinical efficacy of *S. ebulus *5% solution (in ethanol 70%) with that of control, in patients affected by paederus dermatitis for the first time. 

## Experimental

*Methods*

Plant material and extraction *S. ebulus *fruit were collected from Panbe Chooleh, Sari, Iran in September 2010 and confirmed by Dr B. Eslami (Plant systematic specialist). A voucher (No. 135) has been deposited in herbarium (Qaemshahr branch, Islamic Azad University, Qaemshahr, Iran). Fruit were dried at room temperature and coarsely ground before extraction. Six hundered grams of powdered sample was fractionated by successive solvent extraction at room temperature by percolation with hexane (2.4 L × 3) then ethyl acetate (2.4 L × 3) and finally methanol (2.4 L × 3) successively. We have recently improved toxicity in ethyl acetate fraction (2^nd^ fraction) and safety in methanol fraction (3^rd^ fraction) (18). The resulting methanol extract was the concentrated over a rotary vacuum evaporator (30-35°C) until a solid extract sample was obtained. The resulting crude extracts were freeze-dried (Yield: 17%). For preparation of a 5% solution, 5 g of powder was dissolved in 95 g of ethanol (70%) for clinical study. 

*Determination of total phenolic compounds and flavonoid contents*

Total phenolic compound contents were determined by the Folin-Ciocalteau method (25, 26). The extract sample (0.5 mL) was mixed with 2.5 mL of 0.2 N Folin-Ciocalteau reagent for 5 min and 2.0 mL of 75 g l^-1^ sodium carbonate was then added. The absorbance of reaction was measured at 760 nm after 2 h of incubation at room temperature. Results were expressed as gallic acid equivalents. Total flavonoids were estimated using our recently publish papers (25, 26). Briefly, 0.5 mL solution of extract in methanol were mixed with 1.5 mL of methanol, 0.1 mL of 10% aluminum chloride, 0.1 mL of 1 M potassium acetate, and 2.8 mL of distilled water and left at room temperature for 30 minutes. The absorbance of the reaction mixture was measured at 415. Total flavonoid contents were calculated as quercetin from a calibration curve.

*Clinical trial and statistical analysis*

**Figure 2:**
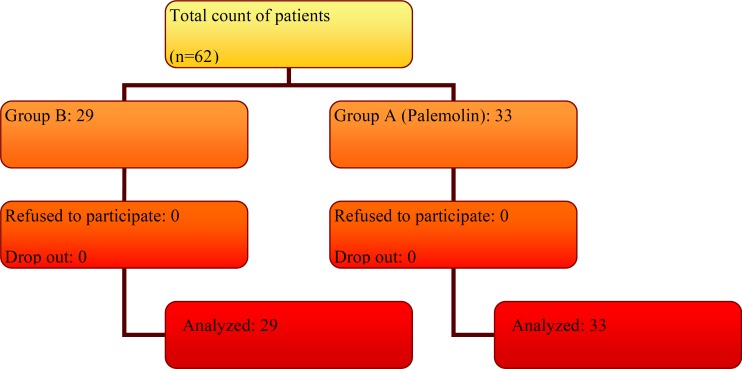
Flowchart of patient enrollment in two groups

This randomized double-blind, prospective, placebo-controlled clinical trial was carried out in the north of Iran (Mazandaran province) during January to September 2011. The protocol was approved by the Institutional Review Board (IRB) of Mazandaran University of Medical Sciences (Grant No. 1389-54). The patients provided informed consent in accordance with the procedures outlined by the local IRB, and were informed that they could withdraw from trial at any time. The trial was performed in accordance with the Declaration of Helsinki and subsequent revisions (27). Their diagnosis was based on clinical sings and physical examination of dermatitis due to paederus beetles lasted less than 24 h of onset of the lesion, lack of previous treatment and age ranged above 10 years. Periorbital involvement and conjunctivitis, pregnant or nursing women and under 10 years children were excluded. Sixty two patients were enrolled by numbers randomized table sampling method and divided in two therapeutic groups A and B (Figure 2). Patients in Group A (33 patients) were treated with a topical solution of palemolin (a 5% *S. ebulus *fruit extract in ethanol 70%) thrice a day and hydrocortisone topical ointment three times daily. Group B (29 patients) were received ethanol 70% topical solution and hydrocortisone topical ointment thrice a day. All medicines were prescribed by a dermatologist as a main colleague of the study and all therapeutic agents prepared in the same shapes by the pharmacist as a co-investigator. Patients were advised to use drugs regularly and do not use other drugs during treatment. All patients were visited or asked every 12 h. Results of each questionnaire were recorded. The effects of drugs in terms of affecting on itching, burning, pain, inflammation, healing rate, drying the wound and infections were studied (28). Four levels, namely, ineffective (value zero), not bad (value one), good effect (value two) and great effect (value three) were chosen for evaluation. For safety evaluation, all adverse events, reported or observed, were recorded at each visit. Routine physical examinations were conducted at each visit. Lesion sizes as a wound parameter were measured in the beginning and during of the treatment. Statistical analysis performed by using SPSS_16 _software and nominal variables were expressed as count (percentage), and statistical analysis of contingency tables was performed by Chi-square and Mann Whitney tests. A p-value of < 0.05 was considered significant.

## Results and Discussion

The extract was standardized by its total phenol and flavonoids contents. The likely most therapeutically important compounds in *Sambucus *spp. are phenols and flavonoids. Assays were performed according to recently published methods (25, 26), Total phenol compounds, as determined by Folin Ciocalteu method, are reported as gallic acid equivalents by reference to standard curve (y= 0.0058x, r^2^= 0.989). The total phenolic content was 93.97 ± 4.7 mg gallic acid equivalent/g of extract. The total flavonoid content was 9.94 ± 0.34 mg quercetin equivalent/g of extract, by reference to standard curve (y= 0.0064x - 0.0076, r^2^= 0.999). Demographic variables including age, sex and residence of patients for two groups were shown in Table 1 and there was no significant difference with regard to these variables, so patients were comparable in two groups. More than half of patients in the treatment (palemolin) group and most of them in control group had 20-40 years old (48.4%) and with male majority (69.4%) but sex distribution were similar in two groups. 

**Table 1:** Demographic characteristics of the study sample of the patients

**Variables**		**Group A ** **No. (%)**	**Group B ** **No. (%)**	**Total** **No. (%)**
Age (year)	<20	4 (12.12)	5 (17.24)	9 (14.52)
	20-40	17 (51.51)	13 (44.83)	30 (48.38)
	>40	12 (36.36)	11 (37.93)	23 (37.1)
Sex	Male	22 (66.66)	21 (72.41)	43 (69.35)
	Female	11 (33.33)	8 (27.58)	19 (30.64)
Residence	Urban	15 (45.45)	14 (48.28)	29 (46.77)
	Rural	18 (54.55)	15 (51.72)	33 (53.22)

Group A were treated with a topical solution of palemolin (a 5% *S. ebulus *fruit extract in ethanol 70%) and hydrocortisone topical ointment three times daily. Group B were treated with ethanol 70% topical solution and hydrocortisone topical ointment thrice a day.

Lesion’s characteristics in patients with paederus dermatitis have been showed in Table 2. In both groups, the most common site of wound was observed in neck and trunk (43.5%) and followed by head and face (37.1%). Legs had the least involvement (6.5%). The lesion sizes were less than 10 cm in 30.6% and in the range of 10-20 cm in 27.4% of patients and control groups. Patients presented with 21-30 and >40 cm^2^ wound surface area were equally (6 patients) in two groups.

**Table 2:** Lesion’s characteristics of patients with paederus dermatitis

	**Variables**	**Group A ** **No. (%)**	**Group B** **No. (%)**	**Total** **No. (%)**
Lesion place	Head and face	13 (39.4)	10 (34.5)	23 (37.09)
	Neck and trunk	13 (39.4)	14 (48.3)	27 (43.54)
	Hands	5 (15.15)	3 (10.3)	8 (12.9)
	Legs	2 (6.06)	2 (6.9)	4 (6.45)
Lesion size (cm^2^)	<10	11 (33.33)	8 (27.58)	19 (30.64)
	10-20	10 (30.30)	7 (24.13)	17 (27.42)
	21-30	6 (18.18)	6 (20.68)	12 (19.35)
	31-40	0 (0)	2 (6.89)	2 (3.22)
	>40	6 (18.18)	6 (20.68)	12 (19.35)

Group A were treated with a topical solution of palemolin (a 5% *S. ebulus *fruit extract in ethanol 70%) and hydrocortisone topical ointment three times daily. Group B were treated with ethanol 70% topical solution and hydrocortisone topical ointment thrice a day.

Therapeutic response parameters in palemolin group (A) and control group (B) were presented in Table 3. Palemolin was statistically more effective in controlling of burning, inflammation, drying the wound, infections and acceleration of healing than control group (p ≤ 0.05). Specially in controlling of inflammation, palemolin had more significant efficacy (p < 0.001) than control group. Only the anti itching effect of palemolin was similar to that of control groups (p = 0.7). Therapeutic outcome in the two groups are shown in Table 4. At least 21 percent of palemolin treated groups were cured at a period of 12 hours (versus 6.7% in control groups). The rate of successful treatment was meaningfully better in group A compare with group B. About 63.6% of patients in palemolin group cured during first 24 h (versus 27.4% in control groups). The problems related to lesions in 93.9% of patients were eliminated completely during 48 hours after the beginning of the treatment by palemolin (versus 65.4% in control groups). Only 2 (6.1%) of patients needed to continue using of drugs more than 48 hr in palemolin treated groups vs. 10 (34.4%) of patients (one/third) in control group. Healing time was statistically faster in palemolin treated patients control groups (p = 0.028).

**Table 3:** Therapeutic response of the patients to treatment methods

**Therapeutic effect on**	**Groups**	**Great effect** **No (%)**	**Good effect ** **No (%)**	**Average effect** **No (%)**	**Ineffective** **No (%)**	**Problem** **No (%)**	**p-value**
Itching	Group A	1(3)	13(39.4)	5(15.2)	2(6.1)	12 (36.4)	0.71
	Group B	0(0.0)	10 (34.5)	7(24.1)	3(10.3)	9 (31)	
Burning	Group A	2(6.1)	10(30.3)	5(15.2)	1( 3)	15(45.5)	0.04
	Group B	0(0.0)	4(13.8)	6 (20.7)	7 (24.1)	12(41.4)	
Pain	Group A	8(24.24)	15(48.5)	5(15.2)	2 (6.1)	3 (9.1)	0.49
	Group B	4(13.8)	10(34.5)	9(31)	2(6.9)	4(13.8)	
Inflammation	Group A	14(42.4)	16(48.5)	1 (3)	1 (3)	1(3)	0.001
	Group B	4(13.1)	9 (31)	14(48.3)	2(6.9)	0 (0.0)	
Wound healing	Group A	14(42.4)	12(36.4)	6(18.2)	1(3)	0(0.0)	0.02
	Group B	4(13.8)	13 (44.8)	10(34.5)	2( 6.9)	0(0.0)	
Wound drying	Group A	18(54.5)	11(33.3)	4(12.1)	0 ( 0.0)	0 (0.0)	0.04
	Group B	8(27.6)	11(37.9)	6 (20.7)	4(13.8)	0 (0.0)	
Infection prophylaxis	Group A	16(48.5)	13(39.4)	4 (12.1)	0 (0.0)	0 (0.0)	0.05
	Group B	8(27.6)	9 (31)	10(34.5)	2( 6.9)	0(0.0)	

Group A were treated with a topical solution of palemolin (a 5% *S. ebulus *fruit extract in ethanol 70%) and hydrocortisone topical ointment three times daily. Group B were treated with ethanol 70% topical solution and hydrocortisone topical ointment thrice a day.

**Table 4:** Therapeutic response of the patients and control groups to the two treatment methods

**Therapeutic improvement time (h)**	**Group A ** **No. (%)**	**Group B** **No. (%)**	**Total** **No. (%)**	**p-value**
12	7 (21.21)	2 (6.84)	9 (14.51)	0.028
24	14 (42.42)	6 (20.68)	20 (32.25)	
36	6 (18.18)	5 (17.24)	11 (17.75)	
48	4 (12.12)	6 (20.65)	10 (16.13)	
60	0 (0)	5 (17.24)	5 (8.06)	
≥72	2 (6.06)	5 (17.24)	7 (11.29)	

Group A were treated with a topical solution of palemolin (a 5% *S. ebulus *fruit extract in ethanol 70%) and hydrocortisone topical ointment three times daily. Group B were treated with ethanol 70% topical solution and hydrocortisone topical ointment thrice a day.

Preventing human/beetle contact is the primary method of avoiding Paederus dermatitis. Learning to recognize paederus beetles and avoiding handling or crushing these insects will help decrease these eruptions. The Paederus beetle lesion could be managed as irritant contact dermatitis-removal of irritant, initial washing with soap and water, application of cold wet compresses followed by topical steroid and antibiotic, if secondarily infected (6). In absence of exclusively treatment, numerous agents were used to manage paederus dermatitis in some studies. An interesting study was performed in Sierra Leone with 36 patients. Half of the patients were given oral ciprofloxacin in addition to a topical steroid. Healing time was statistically faster in these patients, which suggests a concurrent bacterial infection was present, most likely from the *Pseudomonas *the Paederus beetle harbors (29). Good antibacterial activities have been reported from *S. ebulus *(22, 24). 

Zargari *et al. *described paederus dermatitis in northern Iran by a report of 156 cases (1). There were 70 male (45%) and 86 female (55%) patients. Their ages ranged from 6 months to 74 years (mean age 31.3 ± 16.4 years, median age 32 years). The most common sites of involvement in descending order were the face (42.3%), neck (33.3%), upper extremities (23.7%) and periorbital areas and/or conjunctivae (22.4%). In our study, men were affected with paederus dermatitis more than women (69.4 vs. 30.6%). Similar to Zargari′s report, most of the patients were presented with head, face and neck. 

Even oral atorvastatin, a drug that specially use to decrease the serum lipids, were compared with topical steroid in treatment of beetle paederus lesions. Although the authors reported both of triamicinolone ointment and oral atorvastatin has similar effect on Paederus dermatitis, they didn’t recommend atorvastatin to treat this self-limited disease (30). 

The most considerable therapeutic effect of palemolin in this study was its anti-inflammation property. Previous study was demonstrated good anti-inflammatory effect of *S. ebulus *could explain this effect) (16, 23). Inflammation is a common and important component of paederus lesions, so introduction an agent to subside inflammation and other problems related to paederus dermatitis (pain, burning …) is very useful in clinical practice. It seems that palemolin can remove clinical symptoms and signs of paederus dermatitis fast and completely. Based on the results of this study, it is recommended *S. ebulus *5% solution may be considered as an effective preparation in the treatment of paederus dermatitis.
